# Courtship behavior in *Drosophila melanogaster*: towards a ‘courtship connectome’

**DOI:** 10.1016/j.conb.2012.09.002

**Published:** 2013-02

**Authors:** Hania J Pavlou, Stephen F Goodwin

**Affiliations:** Department of Physiology, Anatomy & Genetics, University of Oxford, Oxford, OX1 3PT, UK

## Abstract

The construction of a comprehensive structural, and importantly functional map of the network of elements and connections forming the brain represents the Holy Grail for research groups working in disparate disciplines. Although technical limitations have restricted the mapping of human and mouse ‘connectomes’ to the level of brain regions, a finer degree of functional resolution is attainable in the fruit fly, *Drosophila melanogaster*, due to the armamentarium of genetic tools available for this model organism. Currently, one of the most amenable approaches employed by *Drosophila* neurobiologists involves mapping neuronal circuitry underlying complex innate behaviors – courtship being a classic paradigm. We discuss recent studies aimed at identifying the cellular components of courtship neural circuits, mapping function in these circuits and defining causal relationships between neural activity and behavior.

## Highlights

► *Drosophila* is a useful model for mapping neuronal circuitry underlying sexual behavior. ► We review studies aimed at identifying the cellular components of courtship neural circuits. ► Mapping function in circuits defines causal relationships between neural activity and behavior. ► Optogenetic and thermogenetic strategies have been pivotal for the identification of command elements capable of eliciting courtship.

“*The ascendancy of network science has been driven by the growing realization that the behaviour of complex systems – be they societies, cells or brains – is shaped by interactions among their constituent elements*” – (*Ed Bullmore and Olaf Sporns, 2009*) [1]

**Current Opinion in Neurobiology** 2013, **23**:76–83

This review comes from a themed issue on **Neurogenetics**

Edited by **Ralph Greenspan** and **Christine Petit**

For a complete overview see the Issue and the Editorial

Available online 28th September 2012

0959-4388/$ – see front matter, © 2012 Elsevier Ltd. All rights reserved.

**http://dx.doi.org/10.1016/j.conb.2012.09.002**

## Introduction

Studies on courtship behavior have focused on two pivotal transcription factors of the *Drosophila* sex-determination hierarchy, *fruitless* (*fru*) and *doublesex* (*dsx*). These transcription factors act in concert to specify sex-specific physiology and neural circuitry [2]. Historically, how *fru* and *dsx* regulate courtship came from behavioral analyses in males and females expressing mutations at the respective loci [3]. How these genes function in specifying sexual behavior was inferred by the temporal and spatial patterns of *fru* and *dsx* expression in the nervous system, which facilitated the identification of candidate cellular components of this circuit [2].

To understand circuit organization, it is essential to functionally and neuroanatomically characterize the component parts. Silencing neurons (determining necessity), and artificially triggering the same neurons (determining sufficiency) helps ascertain causal relationships between neurons and behavior. Integrating neuroanatomical and behavioral data for each neuronal class sheds light on how information is received and interpreted, from sensory input through higher-order processing to motor outputs. While sensory neurons and motoneurons may be viewed respectively as the start and end points of this connectome, complex circuit organization arises from interneurons of the CNS that are responsible for higher order processing, and decision-making. This review will focus on studies that have defined patterns of connectivity and/or functionally mapped circuit elements underlying courtship behavior.

## Courtship behavior

*Drosophila* males display a complex repertoire of behaviors that have evolved to achieve reproductive success. This includes following the female, tapping her with his forelegs, contacting her genitalia with his mouthparts, singing a species-specific courtship song, and bending his abdomen to copulate [3]. It is presumed that *Drosophila* females assess a courting male by ‘summating’ sensory cues for species type and fitness before sanctioning mating [3]. A virgin female has the ability to be unreceptive to and resist the courtship of a *Drosophila* male by exhibiting rejection behaviors, which include extruding her ovipositor, kicking, or decamping [3–6]. If she decides to accept the male, she slows down, ceases rejection behaviors and opens her vaginal plate for copulation [3]. After successful copulation, mated females become temporarily sexually unreceptive to further copulatory attempts, increasing their rate of egg-laying [7].

## Circuitry underlying courtship behavior

Expression of *fruitless* and *doublesex* in sensory neurons, interneurons and motorneurons suggests that they are organized into circuit elements capable of receiving, processing and transferring information that controls sexual behavior. Indeed impinging the activity of all, or some, of these neurons have profound effects on male and female courtship behaviors [2]. While male-specific proteins Fru^M^ and Dsx^M^ act in concert to specify male-specific circuitry [8–12], female-specific circuitry appears largely determined by the female-specific *dsx* protein, Dsx^F^ [12]. Given that the neuronal clusters expressing these sex-specific proteins form dimorphic neural networks directing sex-specific behavioral outputs, comparing males and females sheds light on how differing behavioral outputs may be engendered via shared circuits that operate differently and/or sex-specific circuits that result from the presence/absence of unique circuit elements.

Tools that gain genetic access to *fru* and *dsx* neurons have exploited the GAL4/UAS and LexA/lexAop binary systems, and more recently FLP recombinase has been used for genetic access to subsets of neurons [12–15,16^•^,17,18^•^,19^•^,20,21]. A comprehensive digital 3D atlas of *fru* neurons has been achieved using a combination of these tools in two independent approaches [19^•^,22,23]. Warping high-resolution tracings of individual neurons onto a common reference brain allowed the identification of neuro-anatomical dimorphisms and, by implication, differences in interconnectivity and likely pathways of neural processing [18^•^,19^•^]. Direct comparison of male vs. female *fru* neural elements confirmed three distinct modes of sexual dimorphism; (i) sex-specific neuronal clusters; (ii) differences in cell numbers within a given cluster; and (iii) sex-specific arborizations; all of which may contribute to developing neural architecture capable of eliciting sex-specific behavior(s) [11,13–17,18^•^,19^•^,24,25^•^,26^•^]. Although the connectivity between component neurons is largely hypothetical, this *fru* ‘digital atlas’ is serving as a neuroanatomical framework for functional studies.

## Relaying sensory information

**Figure 1:**
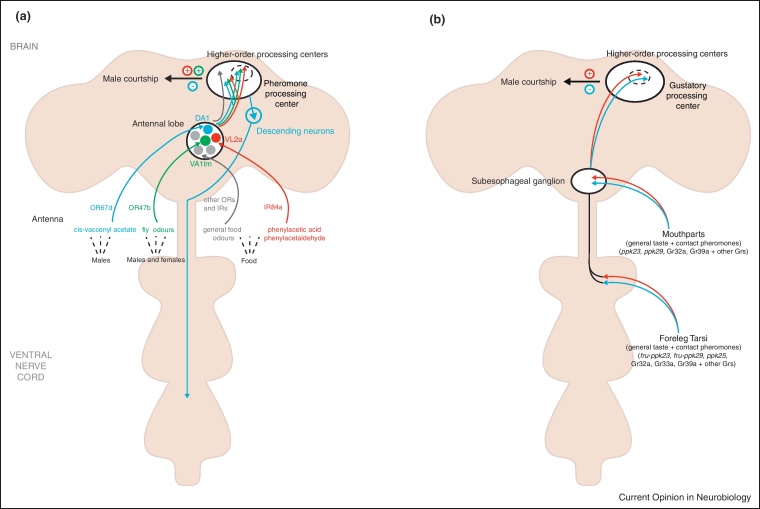
Proposed model for the perception and integration of sensory information in the male CNS. **(a)** Olfaction: Olfactory receptor neurons (ORNs) in the antenna detect odors and send axons to glomeruli of the antennal lobe of the brain. DA1 and VA1lm glomeruli receive pheromonal information from *Or67d*-expressing neurons (which respond to the male pheromone 11-*cis*-vaccenyl acetate, cVA) [24,31] and *Or47b*-expressing neurons (which respond to unidentified female and male-derived odors) [58,59]. While Or67d ORNs express fru, Or47b ORNs do not. Olfactory information is propagated through projection neurons to higher brain centers, such as the mushroom body and lateral horn. OR67d/DA1 neurons target fru-expressing second order neurons in a specific region of the lateral horn associated with pheromone processing. Ultimately these second order neurons form downstream fru-expressing connections (via third and fourth order neurons) that terminate in the abdominal ganglia [24,25^•^]. VL2a glomerulus receives information from *Ir84a*-expressing neurons, which respond to odors derived from host food/oviposition substrates [34]. VL2a projection neurons are segregated from projection neurons responding to general food odor pathways but they are anatomically interconnected with the VA1lm/DA1 pheromone pathways and target a specific area in the lateral horn involved in pheromone processing [34]. Note that only half of the male CNS is shown in the schematic. Adapted from [60]. **(b)** Gustation: In males, non-volatile pheromones are primarily detected by gustatory receptor neurons (GRNs) of the foreleg tarsi. *Gr33a* and *Gr32a* are thought to detect female-specific pheromones (acting as aphrodisiacs) and *Gr39a* is thought to detect male-specific pheromones (acting as aversive stimuli) [38,40,41]. GRNs expressing these Grs do not express fruitless. However, projections of *Gr32a*-expressing terminate in the SOG are likely to form connections with fru-expressing neurons of the ventrolateral protocerebrum that relay this signal to the higher-order lateral protocerebrum [42]. Projections of *Gr33a*-expressing and *Gr39a*-expressing GRNs remain unknown. GRNs expressing proprioceptive receptors *pickpocket-23* (*ppk23*), *ppk29* or *ppk25* co-express fruitless and have also been implicated in courtship [19^•^,44,45,47,48]. All three send sexually dimorphic projections to the first thoracic ganglia, of which *ppk23*-expressing and *ppk29*-expressing GRNs ascend to terminate in the SOG [19^•^,44,45,48]. Although downstream connections have not been described, studies have shown that gustatory information is relayed from the SOG to a distinct region of the lateral protocerebrum [16^•^,19^•^]. One population of *ppk23*-expressing GRNs respond to male-specific pheromones (to promote male–male repulsion) [44], while a second population responds to female-specific pheromones (to promote male–female courtship) [44,45]. Most aforementioned receptors are also expressed in the mouthparts; however, their specific role in these tissues has not been determined. Note that only half of the male CNS is shown in the schematic.

*fru*^*GAL4*^ is expressed in approximately 15% of the olfactory receptor neurons (ORNs) on the third antennal segment, which innervate olfactory trichoid sensilla [14,15]. Blocking synaptic transmission in these ORNs profoundly reduces male courtship, demonstrating their importance in detecting sex-pheromones [15]. The afferent projections of these *fru*^*GAL4*^ ORNs have been mapped to three sexually dimorphic antennal lobe glomeruli; DA1, VA1lm and VL2a, which appear to overlap with dendrites of second order *fru*^*GAL4*^ projection neurons (PNs; Figure 1A) [15,27]. A complete map of higher olfactory centers showed that pheromone-responsive PNs project to distinct compartments of the lateral horn of the protocerebrum, a region thought to be the ‘sensory integration/processing center’ [18^•^,19^•^,28].

To date our knowledge of the neuronal circuitry underlying the detection, processing and possible outputs of olfactory pheromones has come exclusively from studies on the male pheromone 11-*cis*-vaccenyl acetate (cVA) (Figure 1A) [24,25^•^,29]. cVA, sensed by Or67d ORNs in both males and females, elicits aggression amongst males [30–32], inhibits male courtship towards males and females [31,33], and promotes receptivity in females. Mapping the cVA-responsive circuit showed that it comprised as few as four physiologically connected neurons, with the fourth neuron projecting into the ventral nerve cord (VNC) and terminating in the thoracic abdominal ganglia (Abg; Figure 1A) [25^•^]. While some components within the circuit are male-specific others exhibit male-specific arborizations and/or synaptic connections [24,25^•^]. Given that dimorphic anatomical connections are particularly enriched in third order olfactory interneurons of the lateral horn [18^•^], these neurons may be the first elements in the olfactory pathway that respond sex-specifically to the same stimulus. In particular, dimorphic connections between DA1 PNs and higher-order olfactory neurons might explain the sex-specific behavioral responses elicited by cVA [18^•^,25^•^], though additional electrophysiology experiments are essential to determine this.

While the association between pheromones, olfaction and courtship may seem obvious, an intriguing relationship has been identified between food-olfaction and male courtship [34]. Members of a novel family of chemosensory receptors, the ionotropic glutamate receptors (Irs), localize to distinct sensory cilia of the antennae and are expressed in olfactory neurons lacking olfactory receptors (ORs) [35]. In particular, ORNs that express *Ir84a* have been shown to co-express *fru*^*M*^ and innervate VL2a, one of the three *fru*^+^ sexually dimorphic glomeruli (Figure 1A) [34]. Although *Ir84a*^+^ neurons are not tuned to respond to sex-specific odors, two volatile compounds normally found in *Drosophilid* food sources (phenylacetaldehyde and phenylacetic acid) were found to elicit responses in both males and females. *Ir84a* mutant males court females at diminished levels, while restoring *Ir84a* function re-establishes normal courtship levels, implicating *Ir48a* in the regulation of male courtship. Neuroanatomical analyses of second order VL2a PNs revealed distinctive intercalation with pheromonal PNs of *fru*^+^ DA1 and VA1lm glomeruli, rather than with general food odor pathways [34] (Figure 1A). PNs from all three sexually dimorphic glomeruli target a specialized pheromone-processing region of the lateral horn, suggesting a point of integration of food and pheromonal pathways within the *fru*^+^ ‘courtship connectome’.

During the ‘tapping’ and ‘licking’ stages of courtship males are believed to assess a mate's sex and species via non-volatile pheromones, with the reception of essential information occurring largely via gustatory receptor neurons (GRN) of the foreleg (Figure 1B) [36]. Studies suggest that detection of female-enriched long-chain cuticular hydrocarbons (CHCs) stimulates male–female courtship while the detection of male-enriched CHCs promotes male–male repulsion, preventing inappropriate male–male courtship [37]. To date, only three gustatory receptors (Gr) have been implicated in the reception of pheromones – *Gr39a* is thought to detect to female-enriched CHCs [38], while *Gr32a* and *Gr33a* may serve to detect male-enriched CHCs [39–41]. GRNs expressing each of these Grs do not express *fru*. However, axons of *Gr32a*-expressing GRNs terminate in the SOG and are likely to form connections with second-order *fru*-expressing neurons of the ventrolateral protocerebrum that relay this signal to the higher-order lateral protocerebrum (Figure 1B) [42]. This implies that sensory neurons that do not express *fru* may be capable of relaying information to *fru*^+^ downstream ‘processing’ circuitry.

Members of the degenerin/epithelial sodium channel subunit family *pickpocket-23* (*ppk23*) and *pickpocket-29* (*ppk29*) are expressed in Fru^M^-positive GRNs of the foreleg tarsi [43–45]. There appears to be twice the number of *ppk23*^+^ neurons in the male foreleg, when compared to the female [43–45], with contralateral projections within the VNC that terminate in the SOG, a sex-specific feature previously attributed to Fru^M^-expressing gustatory neurons (Figure 1B) [17,46]. Males with silenced *ppk23*^+^ neurons display reduced levels of courtship towards females and increased levels towards males, while males with silenced *ppk29*^+^ neurons display defects in courtship towards females only [43–45]. Coupling thermogenetics with electrophysiology demonstrated that *ppk23*^+^ neurons, which are restricted to two cells per bristle, respond to specific female pheromones [44,45]. G-CaMP imaging of *ppk23*^+^ neurons in response to pheromonal stimulation showed that the two neurons innervating each bristle perform opposing roles forming two directly competing populations; one specifically responding to male-enriched pheromones, the second responding to female-enriched pheromones, suggesting a direct role in mediating attractive and aversive cues [44]. A third subunit, *pickpocket-25*, is expressed in sexually dimorphic *fru*^+^ tarsal gustatory neurons that also give rise to characteristic sexually dimorphic projections within the VNC (Figure 1B). Though these neurons appear to be necessary for normal courtship initiation, specific ligands and further neuronal connections remain unknown [47,48].

**Figure 2:**
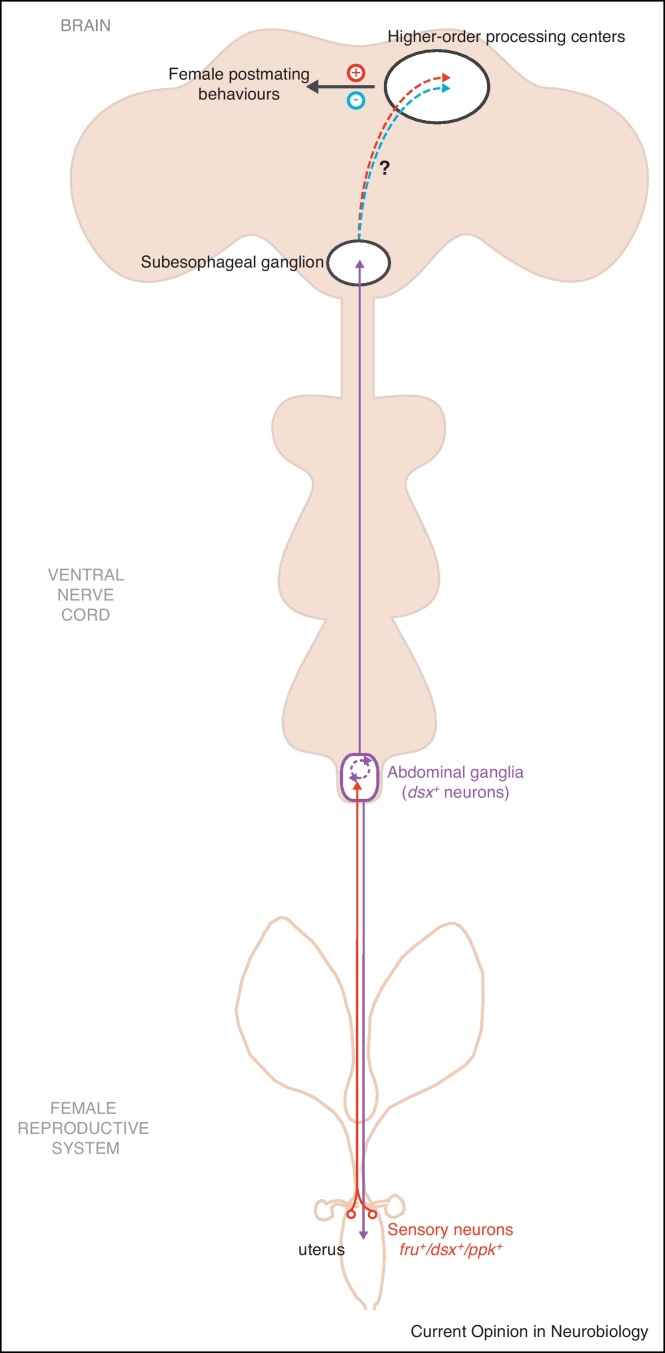
Proposed model for the perception and integration of sex peptide (SP) sensory signals that regulate female postmating responses. SP-responsive sensory neurons expressing *fru*, *dsx* and *ppk* are localized to the uterus and project into the abdominal ganglia (Abg) and possibly into the suboesophageal ganglion (SOG) [21,49,50]. A cluster of *dsx*-expressing neurons of the Abg is required for the induction and regulation of specific postmating responses [21]. This cluster consists of (1) ascending neurons innervating the central brain, (2) descending neurons innervating the uterus and (3) local interneurons potentially (dotted line) conveying information to/from central circuits regulating female mating decisions. Higher-order central pathways may process these signals (dotted lines) to regulate female behavior; however, studies have yet to identify these processing/command elements. Note that only half of the female CNS is shown in the schematic.

Adapted from [60].

The neuronal basis of female sexual behaviors has only recently come into play with studies primarily focusing on sensory elements that modulate postmating responses (Figure 2) [21,49,50]. Postmating responses are triggered primarily by the allohormone pheromone sex peptide (SP), a small peptide synthesized in the male accessory glands that is transferred to the female during insemination [7]. A SP-responsive G protein-coupled receptor for SP-mediated postmating responses has been identified; females lacking this sex peptide receptor (SPR) remain receptive, exhibiting virgin-like behaviors, postmating [51]. Transfer of male sex peptide (SP) during copulation mediates these postmating responses by SPR activation in sensory neurons that coexpress *fru*, *dsx* and the proprioceptive neuronal marker *pickpocket* (*ppk*) in the female reproductive system [21,49,50]. In addition, a sex-specific cluster of *dsx*-expressing neurons in the Abg are required for the induction and regulation of some postmating responses [21]. This cluster consists of (1) interneurons that form synaptic arborizations within the SOG that may act downstream of sensory elements of the uterus to convey sensory information to higher-order centers, (2) neurons that form presynaptic arborizations on the uterus that may be involved in directing motor output, and (3) local interneurons that may regulate both (Figure 2).

## Deciding to court

Determining causal relationships between neurons and behavior, Clyne and Miesenböck (2008) used an optogenetic strategy to activate all *fru*-expressing neurons in intact and headless flies [52]. They demonstrated the existence of a central song pattern generator (CPG) in the mesothoracic area of the VNC in both sexes, capable of generating wing extension and courtship song in flies. However, to generate bona fide courtship song requires the expression of the male-specific isoforms of both *dsx* (Dsx^M^) and *fru* (Fru^M^) in the CNS, in addition to direction from male-specific higher-order ‘command’ neurons [9,52].

**Figure 3:**
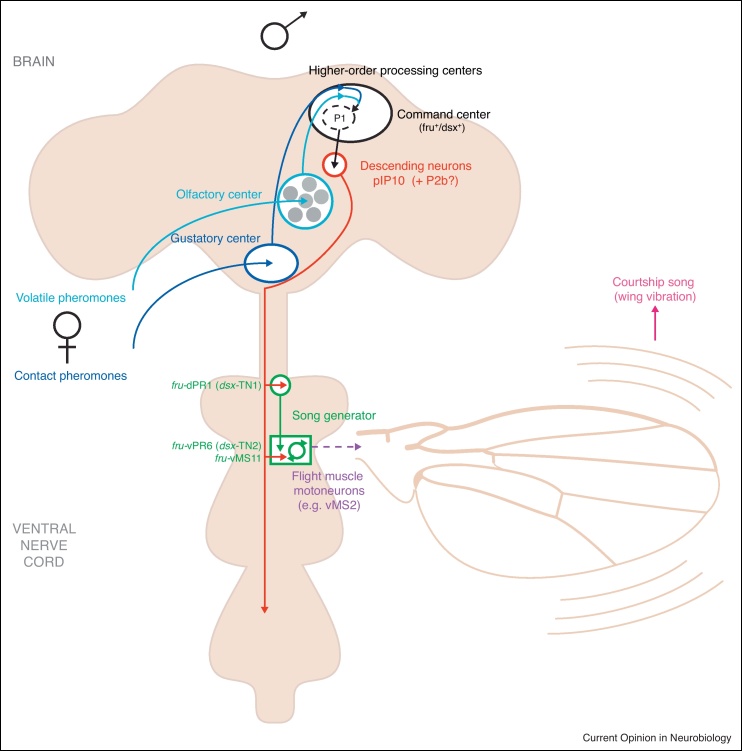
Proposed circuitry underlying the initiation of courtship and pulse song in the male CNS. During courtship, a male extends and vibrates his wing to sing a species-specific courtship song. Five distinct fru-expressing clusters of neurons that consist of command/decision-making neurons (P1), descending interneurons (pIP10), and components of the central pattern generator (dPR1, vPR6 and vMS11) are thought to form an interconnected circuit [26^•^]. Thermoactivation of these neurons triggers wing extension and/or vibration in isolated males deprived of the sensory inputs that would normally induce males to sing [26^•^]. Thermoactivation of P1 and the descending neuron P2b independently initiate male courtship in the presence of a female. Sensing volatile and/or contact pheromones subsequently alters the activity of the P1 cluster, suggesting its capability of integrating olfactory and gustatory sensory cues when making the decision to court. Thus P1 and P2b are thought to play a key role in triggering male courtship [53^••^]. Although the descending cluster, P2b, is connected to P1 neurons its downstream connections within the VNC have yet to be identified. A cluster of flight muscle motoneurons, vMS2, may (dotted line) form connections with the CPG [19^•^]. Note that only half of the male CNS is shown in the schematic.

Adapted from [61].

Continuing with this logic, that higher-order command neurons exist that initiate courtship behaviors, Kimura *et al.* (2008) identified a *fru*/*dsx*-expressing neuronal cluster in the dorsal posterior brain, P1, as an important neuronal element that can initiate male-type courtship behavior [11]. While restrictively thermoactivating the P1 cluster is sufficient to initiate courtship and trigger pulse song, in the presence or absence of a female [26^•^,53^••^], silencing P1 neurons in the male brain impairs song and other courtship elements [11,26^•^,53^••^]. Since P1 neurons are male-specific and are located in the lateral protocerebrum (which receives multimodal sensory input), they are ideal ‘decision-making’ candidates capable of integrating environmental stimuli to make the decision to court (Figure 3). A remarkable experiment demonstrated that while contact between the male tarsus and a female's abdomen provokes real-time activation of P1 neurons, the presence of cVA attenuates this response [53^••^], demonstrating a role for P1 neurons in integrating gustatory and olfactory information. In line with this, Pan *et al.* (2012) showed that restricted activation of P1 neurons resulted in heightened levels of following and orienting when exposed to a moving object [54]. Although visual processing appears independent of the P1 neuronal pathway, these data support the view that, by integrating multimodal sensory signals, P1 interneurons mediate the decision to initiate courtship, effectively acting as a switch for courtship initiation [26^•^,53^••^,54].

Thermoactivation of *fru*^+^ descending interneurons, P2b and pIP10, is sufficient to initiate courtship and trigger pulse song, in the presence or absence of a female [26^•^,53^••^]. However, it has been hypothesized that these serve a downstream ‘command’ role, relaying information from integration/higher-processing centers to motor centers in the VNC [26^•^,53^••^]. Within the VNC, three distinct *fru*^+^ interneuron classes are thought to be components of the CPG, as thermoactivation of these neurons did not lead to faithful recapitulation of pulse song such that (1) activation of the *fru*^+^/*dsx*^+^ prothoracic, dPR1, neuron elicits courtship song with a significantly longer inter-pulse interval (IPI), (2) the level of activation of the *fru*^+^/*dsx*^+^ mesothoracic neuronal cluster, vPR6, appears to be indirectly correlated to IPI and (3) activation of the *fru*^+^ mesothoracic neuronal cluster, vMS11, induces wing extension without the production of song [26^•^]. By overlapping axonal and dendritic arborizations, von Philipsborn *et al.* (2011) described a hypothetical interconnected circuit for courtship song, linking all 5 aforementioned *fru*^+^ neuronal classes (P1, pIP10, dPR1, vPR6 and vMS11; Figure 3) [26^•^]. Yu *et al.* (2011) were able to neuroanatomically map a single mesothoracic cluster, vMS2, of *fru*-expressing motoneurons innervating direct flight muscles, which could be critical for song production [19].

## Conclusions

Integrating neuroanatomical data with behavioral data for each neuronal class allows us to generate hypotheses about how information may flow through the system from sensory input, through brain to motor outputs pathways. Although many of the neurons involved in courtship behavior have been identified, additional elements that mediate these signals, and the neural circuits that interpret this information still need to be identified. It is likely that sensory information from all modes of input (visual, taste, smell and hearing) converge within specific brain areas; however, we have no knowledge of how processing centers of the CNS are capable of assigning ‘weighting’ effects associated with any competing agonistic and antagonistic stimuli, to ultimately relay the correct information and elicit most appropriate behavioral outputs. The use of new technology, like optogenetics and thermogenetics [55] has been pivotal for the identification of command elements capable of eliciting courtship and song. These methods of manipulating a defined neural circuit through artificial stimulation complement other new optogenetic techniques, such as those that interrogate physiological connections between component neurons [56]. In future studies, the use of such strategies will allow the marrying of circuit architecture and underlying cellular and synaptic properties to further elucidate how neural pathways control behaviors. So what next? Pinching the logic from Olsen and Wilson's review [57], “Cracking neural circuits in a tiny brain”, we still need to:

• Identify all the neurons that participate in sex-specific behaviors (functional map).
• Establish the ‘natural’ response/activity pattern of all neurons during presentation of a given stimulus and/or execution of a precise behavior (activity map).
• Determine the cellular, synaptic and circuit mechanisms underlying these neural transformations (connectivity map).

Ultimately, correlating the ‘connectivity’, ‘functional’ and ‘activity’ maps will provide the logic for how this, and other, dimorphic nervous systems might be coordinated with gender-appropriate physiology and sensorimotor tissues to generate sex-specific behavioral outputs. Only then may we begin to shed light on the relative values associated with each mode of sensory input, and identify compensatory mechanisms that may exist to allow for behavioral flexibility. Extrapolating models of circuit-organization to define how decisions are achieved to prioritize behavioral outputs in response to conflicting signals, and how experience may modify these behaviors, will enrich our understanding of the astonishing plasticity of nervous systems.

## References and recommended reading

Papers of particular interest, published within the period of review, have been highlighted as:

• of special interest
•• of outstanding interest

## References

[1] Bullmore E., Sporns O. (2009). Complex brain networks: graph theoretical analysis of structural and functional systems. Nat Rev Neurosci.

[2] Dauwalder B. (2011). The roles of fruitless and doublesex in the control of male courtship. Int Rev Neurobiol.

[3] Villella A., Hall J.C. (2008). Neurogenetics of courtship and mating in *Drosophila*. Adv Genet.

[4] Connolly K., Cook R.M. (1973). Rejection responses by female *Drosophila melanogaster*: their ontogeny, causality and effects upon the behavior of the courting male. Behavior.

[5] Spieth H.T., Ringo J.M., Ashburner M., Carson H.J.T. (1983). Mating behavior and sexual isolation in *Drosophila*.

[6] Ejima A., Nakayama S., Aigaki T. (2001). Phenotypic association of spontaneous ovulation and sexual receptivity in virgin females of *Drosophila melanogaster* mutants. Behav Genet.

[7] Kubli E. (2003). Sex-peptides: seminal peptides of the *Drosophila* male. Cell Mol Life Sci.

[8] Billeter J.-C., Rideout E.J., Dornan A.J., Goodwin S.F. (2006). Control of male sexual behavior in *Drosophila* by the sex determination pathway. Curr Biol.

[9] Rideout E.J., Billeter J.-C., Goodwin S.F. (2007). The sex-determination genes fruitless and doublesex specify a neural substrate required for courtship song. Curr Biol.

[10] Sanders L.E., Arbeitman M.N. (2008). Doublesex establishes sexual dimorphism in the *Drosophila* central nervous system in an isoform-dependent manner by directing cell number. Dev Biol.

[11] Kimura K.-I., Hachiya T., Koganezawa M., Tazawa T., Yamamoto D. (2008). Fruitless and doublesex coordinate to generate male-specific neurons that can initiate courtship. Neuron.

[12] Rideout E.J., Dornan A.J., Neville M.C., Eadie S., Goodwin S.F. (2010). Control of sexual differentiation and behavior by the doublesex gene in *Drosophila melanogaster*. Nat Neurosci.

[13] Billeter J.-C., Goodwin S.F. (2004). Characterization of *Drosophila* fruitless-gal4 transgenes reveals expression in male-specific fruitless neurons and innervation of male reproductive structures. J Comp Neurol.

[14] Manoli D.S., Foss M., Villella A., Taylor B.J., Hall J.C., Baker B.S. (2005). Male-specific fruitless specifies the neural substrates of *Drosophila* courtship behaviour. Nature.

[15] Stockinger P., Kvitsiani D., Rotkopf S., Tirián L., Dickson B.J. (2005). Neural circuitry that governs *Drosophila* male courtship behavior. Cell.

[16•] Kimura K., Ote M., Tazawa T., Yamamoto D. (2005). Fruitless specifies sexually dimorphic neural circuitry in the *Drosophila* brain. Nature.

[17] Mellert D.J., Knapp J.-M., Manoli D.S., Meissner G.W., Baker B.S. (2010). Midline crossing by gustatory receptor neuron axons is regulated by fruitless, doublesex and the Roundabout receptors. Development.

[18•] Cachero S., Ostrovsky A.D., Yu J.Y., Dickson B.J., Jefferis G.S.X.E. (2010). Sexual dimorphism in the fly brain. Curr Biol.

[19•] Yu J.Y., Kanai M.I., Demir E., Jefferis G.S.X.E., Dickson B.J. (2010). Cellular organization of the neural circuit that drives *Drosophila* courtship behavior. Curr Biol.

[20] Robinett C.C., Vaughan A.G., Knapp J.-M., Baker B.S. (2010). Sex and the single cell. II. There is a time and place for sex. PLoS Biol.

[21] Rezaval C., Pavlou H.J., Dornan A.J., Chan Y.B., Kravitz E.A., Goodwin S.F. (2012). Neural circuitry underlying *Drosophila* female postmating behavioral responses. Curr Biol.

[22] Lee T., Luo L. (1999). Mosaic analysis with a repressible cell marker for studies of gene function in neuronal morphogenesis. Neuron.

[23] Liu W., Hou S.X. (2008). Genetic tools used for cell lineage tracing and gene manipulation in *Drosophila* germline stem cells. Methods Mol Biol.

[24] Datta S.R., Vasconcelos M.L., Ruta V., Luo S., Wong A., Demir E., Flores J., Balonze K., Dickson B.J., Axel R. (2008). The *Drosophila* pheromone cVA activates a sexually dimorphic neural circuit. Nature.

[25•] Ruta V., Datta S.R., Vasconcelos M.L., Freeland J., Looger L.L., Axel R. (2010). A dimorphic pheromone circuit in *Drosophila* from sensory input to descending output. Nature.

[26•] von Philipsborn A.C., Liu T., Yu J.Y., Masser C., Bidaye S.S., Dickson B.J. (2011). Neuronal control of *Drosophila* courtship song. Neuron.

[27] Kondoh Y., Kaneshiro K.Y., Kimura K.-I., Yamamoto D. (2003). Evolution of sexual dimorphism in the olfactory brain of Hawaiian *Drosophila*. Proc Biol Sci.

[28] Jefferis G.S., Potter C.J., Chan A.M., Marin E.C., Rohlfing T., Maurer C.R., Luo L. (2007). Comprehensive maps of *Drosophila* higher olfactory centers: spatially segregated fruit and pheromone representation. Cell.

[29] Butterworth F.M. (1969). Lipids of *Drosophila*: a newly detected lipid in the male. Science.

[30] Ha T.S., Smith D.P. (2006). A pheromone receptor mediates 11-*cis*-vaccenyl acetate-induced responses in *Drosophila*. J Neurosci.

[31] Kurtovic A., Widmer A., Dickson B.J. (2007). A single class of olfactory neurons mediates behavioural responses to a *Drosophila* sex pheromone. Nature.

[32] Wang L., Anderson D.J. (2010). Identification of an aggression-promoting pheromone and its receptor neurons in *Drosophila*. Nature.

[33] Ejima A., Smith B.P., Lucas C., van der Goes van Naters W., Miller C.J., Carlson J.R., Levine J.D., Griffith L.C. (2007). Generalization of courtship learning in *Drosophila* is mediated by cis-vaccenyl acetate. Curr Biol.

[34] Grosjean Y., Rytz R., Farine J.P., Abuin L., Cortot J., Jefferis G.S., Benton R. (2011). An olfactory receptor for food-derived odours promotes male courtship in *Drosophila*. Nature.

[35] Benton R., Vannice K.S., Gomez-Diaz C., Vosshall L.B. (2009). Variant ionotropic glutamate receptors as chemosensory receptors in *Drosophila*. Cell.

[36] Spieth H.T. (1974). Courtship behavior in *Drosophila*. Annu Rev Entomol.

[37] Ferveur J.F., Cobb M., Bagnères A.G., Blomquist G.J. (2010). Behavioral and Evolutionary Roles of Cuticular Hydrocarbons in *Drosophila*.

[38] Watanabe K., Toba G., Koganezawa M., Yamamoto D. (2011). *Gr39a*, a highly diversified gustatory receptor in *Drosophila*, has a role in sexual behavior. Behav Genet.

[39] Lacaille F., Hiroi M., Twele R., Inoshita T., Umemoto D., Manière G., Marion-Poll F., Ozaki M., Francke W., Cobb M. (2007). An inhibitory sex pheromone tastes bitter for *Drosophila* males. PLoS ONE.

[40] Miyamoto T., Amrein H. (2008). Suppression of male courtship by a *Drosophila* pheromone receptor. Nat Neurosci.

[41] Moon S.J., Lee Y., Jiao Y., Montell C. (2009). A *Drosophila* gustatory receptor essential for aversive taste and inhibiting male-to-male courtship. Curr Biol.

[42] Koganezawa M., Haba D., Matsuo T., Yamamoto D. (2010). The shaping of male courtship posture by lateralized gustatory inputs to male-specific interneurons. Curr Biol.

[43] Lu B., LaMora A., Sun Y., Welsh M.J., Ben-Shahar Y. (2012). *ppk23*-Dependent chemosensory functions contribute to courtship behavior in *Drosophila melanogaster*. PLoS Genet.

[44] Thistle R., Cameron P., Ghorayshi A., Dennison L., Scott K. (2012). Contact chemoreceptors mediate male–male repulsion and male–female attraction during *Drosophila* courtship. Cell.

[45] Toda H., Zhao X., Dickson B.J. (2012). The *Drosophila* female aphrodisiac pheromone activates *ppk23*(+) sensory neurons to elicit male courtship behavior. Cell Rep.

[46] Possidente D.R., Murphey R.K. (1989). Genetic control of sexually dimorphic axon morphology in *Drosophila* sensory neurons. Dev Biol.

[47] Lin H., Mann K.J., Starostina E., Kinser R.D., Pikielny C.W. (2005). A *Drosophila* DEG/ENaC channel subunit is required for male response to female pheromones. Proc Natl Acad Sci USA.

[48] Starostina E., Liu T., Vijayan V., Zheng Z., Siwicki K.K., Pikielny C.W. (2012). A *Drosophila* DEG/ENaC subunit functions specifically in gustatory neurons required for male courtship behavior. J Neurosci.

[49] Häsemeyer M., Yapici N., Heberlein U., Dickson B.J. (2009). Sensory neurons in the *Drosophila* genital tract regulate female reproductive behavior. Neuron.

[50] Yang C.-H., Rumpf S., Xiang Y., Gordon M.D., Song W., Jan L.Y., Jan Y.-N. (2009). Control of the postmating behavioral switch in *Drosophila* females by internal sensory neurons. Neuron.

[51] Yapici N., Kim Y.-J., Ribeiro C., Dickson B.J. (2008). A receptor that mediates the post-mating switch in *Drosophila* reproductive behaviour. Nature.

[52] Clyne J.D., Miesenböck G. (2008). Sex-specific control and tuning of the pattern generator for courtship song in *Drosophila*. Cell.

[53••] Kohatsu S., Koganezawa M., Yamamoto D. (2011). Female contact activates male-specific interneurons that trigger stereotypic courtship behavior in *Drosophila*. Neuron.

[54] Pan Y., Meissner G.W., Baker B.S. (2012). Joint control of *Drosophila* male courtship behavior by motion cues and activation of male-specific P1 neurons. Proc Natl Acad Sci USA.

[55] Bernstein J.G., Garrity P.A., Boyden E.S. (2012). Optogenetics and thermogenetics: technologies for controlling the activity of targeted cells within intact neural circuits. Curr Opin Neurobiol.

[56] Yao Z., Macara A.M., Lelito K.R., Minosyan T.Y., Shafer O.T. (2012). Analysis of functional neuronal connectivity in the *Drosophila* brain. J Neurophysiol.

[57] Olsen S.R., Wilson R.I. (2008). Cracking neural circuits in a tiny brain: new approaches for understanding the neural circuitry of *Drosophila*. Trends Neurosci.

[58] van der Goes van Naters W., Carlson J.R. (2007). Receptors and neurons for fly odors in *Drosophila*. Curr Biol.

[59] Vosshall L.B., Wong A.M., Axel R. (2000). An olfactory sensory map in the fly brain. Cell.

[60] Rezaval C., Fabre C.C., Goodwin S.F. (2011). Invertebrate neuroethology: food play and sex. Curr Biol.

[61] Benton R. (2011). Decision making: singin’ in the brain. Neuron.

